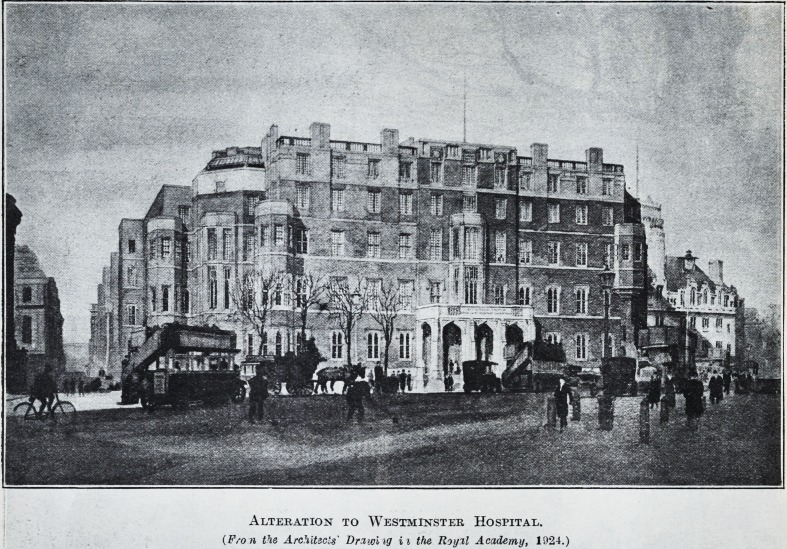# A Transformed Hospital

**Published:** 1924-08

**Authors:** 


### A TRANSFORMED HOSPITAL.

Visitors to Westminster Hospital on July 15 found it transformed almost beyond recognition. It is true that its spotless exterior came as no surprise to those who have watched its coat of London grime slowly disappear, but the bright, airy interior, with cool green walls and spacious corridors, seemed somehow a different place from the rather shabby and antiquated institution, beloved nevertheless by so many. But they will not value it less in its new state, for going through its compact, homely wards, fragrant with flowers sent by the chairman, Sir Edward Pearson, it did indeed seem to be the very model was named in memory of his wife by Mr. James Hora. Here there are the most fascinating pictures in tiles, depicting old favourites of the nursery, such as Goody Twoshoes and Goosey Gander. All this admirable work has been accomplished by the three architects working in co-operation—Messrs. H. Percy Adams, Charles Holden, and Lionel Pearson, all Fellows of the Royal Institute of British Architects.

The Church has ever been the patron of hospitals, and a non-juring priest, Patrick Cockburn, was indeed one of the four original founders of Westmister Hospital. It was natural, therefore, that

Alteration to Westminster Hospital.

(*From the Architects' Drawing in the Royal Academy,* 1924.)

of an abode of healing. The whole hospital community, whether it be the medical staff or the nurses with their pleasant sitting-room and comfortable little bedrooms, the patients with their cheerful wards small enough to be cosy, the fortunate paying patients so charmingly provided for has been equally remembered. The accommodation for private patients is particularly attractive, and is one of the new features at Westminster. On the fourth floor, and compensated for the altitude by the good view and comparative seclusion, some of the rooms will hold four patients, others only one. Each has the same inviting armchair, dainty furniture, curtains, cushions and rug, all of soft, deep blue.

There are new labour and maternity wards, a new operating theatre, new ear, nose and throat wards, new septic wards (male and female), and new eye wards. An example of an increased realisation of the value of beauty in hospital surroundings may be seen in the Marie Celeste Ward for children, which representatives of all sides of the hospital's life should be present at a "Solemn Service of Thanksgiving with Commemoration of Founders and Benefactors," held in the Abbey, under whose shadow it has grown up. Headed by the Porter, these representatives passed in procession to the Abbey, where a simple but very beautiful service was held. After hymns, prayers and anthem—"God's tender mercy knows no bounds"—the Dean, supported by the Abbey clergy, went to the Sacrarium steps and proceeded to read "The Commemoration of Founders and Benefactors." It was a long list containing many illustrious names. A solemn *Te Deum*, Blessing and National Anthem concluded a service at which supporters of the hospital joined with its workers in thanks for the past and hope for the future. So its task of renewal complete, this ancient and admirable institution will on August 1 again throw open its doors to the many who are patiently waiting for its aid.

## Figures and Tables

**Figure f1:**